# No Difference in Ligament Discontinuity Versus Thickening on Magnetic Resonance Imaging When Evaluating Anterior Talofibular Ligament Injuries: A Systematic Review

**DOI:** 10.1016/j.asmr.2025.101194

**Published:** 2025-06-02

**Authors:** Joshua Taylor, Damon V. Briggs, Albert T. Anastasio, Julia E. Ralph, Emily Poehlein, Cynthia L. Green, Stephanie Hendren, Brian C. Lau

**Affiliations:** a UNC School of Medicine, Chapel Hill, North Carolina, U.S.A.; b Duke University School of Medicine, Durham, North Carolina, U.S.A.; c Duke Department of Orthopaedic Surgery, Durham, North Carolina, U.S.A.; d Department of Biostatistics and Bioinformatics, Duke University School of Medicine, Durham, North Carolina, U.S.A.

## Abstract

**Purpose:**

To systematically review the literature evaluating whether incorporating ligament thickening and increased signal intensity alongside ligament discontinuity on magnetic resonance imaging (MRI) improves diagnostic accuracy for anterior talofibular ligament (ATFL) injuries.

**Methods:**

Two independent reviewers conducted a systematic literature search on the basis of Preferred Reporting Items for Systematic Reviews and Meta-Analyses guidelines, using the MEDLINE, Embase, CINAHL Complete, and Scopus databases to find studies reporting MRI and ultrasound imaging findings after ATFL injuries. Statistical analyses were conducted via Review Manager, and a *P* value of <.05 was statistically significant.

**Results:**

In total, 15 studies met inclusion criteria, and 9 provided sufficient data for meta-analyses. MRI demonstrated high sensitivity and specificity for diagnosing ATFL injuries. There were greater diagnostic results for ligament thickening with increased signal intensity, but there was no statistically significant difference between diagnostic approaches on the basis solely of ligament discontinuity and the thickening with increased signal intensity. Pooled sensitivity ranged from 84.7% to 87.1%, whereas specificity ranged from 84.6% to 91.4%. Diagnostic odds ratios were consistently high across methods.

**Conclusions:**

This study found that incorporating ligament thickening and increased signal intensity alongside ligament discontinuity on MRI does not significantly improve diagnostic accuracy.

**Level of Evidence:**

Level III, systematic review of Level II and III studies.

Ankle injuries are among the most common musculoskeletal injuries, particularly in sports.^1^ The anterior talofibular ligament (ATFL) is the most frequently injured ligament in the ankle.^2^^,^^3^ ATFL injuries can result in chronic ankle instability (CAI), recurrent sprains, and long-term joint degeneration if they are not treated appropriately.^4^^,^^5^ Accurate imaging plays an important role in diagnosing ATFL injuries and guiding treatment strategies.^6^

Magnetic resonance imaging (MRI) is extensively used for the diagnosis of ATFL injuries. However, its accuracy and reliability exhibit variability across different studies.^7^^,^^8^ A challenge in MRI interpretation is distinguishing between acute and chronic ATFL injuries. Chronic injuries may exhibit ligament thickening or morphologic changes.^2^^,^^4^ Ligament discontinuity is a primary feature evaluated for in ATFL injuries, with recent studies indicating that alterations in signal intensity and ligament thickening offer diagnostic benefits.^9^^,^^10^ There is currently no standardized protocol on the inclusion of these factors in routine MRI assessments.^5^^,^^11^ The uncertainties raise concern about the misdiagnosis of ligament injuries that could potentially result in mismanagement. This uncertainty impacts clinical management by underestimation of injury severity that can lead to delayed appropriate treatment.

Addressing these gaps are important for a more precise MRI-based diagnosis that can optimize patient outcomes with ATFL injuries. Previous research has evaluated the sensitivity and specificity of MRI compared with arthroscopic findings, but other studies have lacked a standardized methodology that considered subtle characteristics such as signal intensity and ligament thickening.^2^^,^^5^ Thus, an investigation into specific imaging markers, such as ligament thickening and heightened signal intensity in conjunction with ligament discontinuity, on MRI is needed to enhance clinical decision-making, decrease misdiagnosis rates, and improve patient outcomes by refining MRI diagnostic criteria. The purpose of this study was to systematically review the literature evaluating whether incorporating ligament thickening and increased signal intensity alongside ligament discontinuity on MRI improves diagnostic accuracy for ATFL injuries. We hypothesized that the association of ligament thickening and increased signal intensity together with ligament discontinuity on MRI would contribute to the diagnostic precision of ATFL injury detection compared with arthroscopy as a gold standard.

## Methods

### Study Selection

This systematic review was conducted by 2 independent reviewers (J.T. and D.B.) who followed the Preferred Reporting Items for Systematic Reviews and Meta-Analyses guidelines and analyzed the corresponding search results. A senior author (A.A.) arbitrated any disagreements when applicable.^12^ The titles and abstracts identified during the search were screened in a double-blind manner, and potentially eligible studies underwent a full-text review.

### Search Strategy

The databases MEDLINE (via PubMed), Embase (Elsevier), CINAHL Complete (EBSCOhost), and Scopus (Elsevier) were searched for peer-reviewed literature published between 1983 and March 2, 2023, to ensure we have a comprehensive and unbiased collection of the literature. The search algorithm was the following: (anterior talofibular ligament) AND (diagnostic imaging or magnetic resonance imaging or ultrasound or arthrography). Language, or publication filters were added to the search to ensure comprehensiveness.

### Inclusion Criteria

The inclusion criteria involved (1) ATFL injuries, (2) MRI and ultrasound imaging, (3) human and radiographic studies, (4) published in a peer-reviewed journal, and (5) published in English. The exclusion criteria entailed the following: (1) review studies, (2) cadaveric studies, (3) biomechanical studies, (4) case reports, and (5) abstract only.

### Data Collection

All relevant information was collected by 2 independent reviewers (J.T. and D.B.) using a predetermined data sheet on Microsoft Excel. When required information was not available in the text, the authors were contacted via email. The level of evidence was assessed using the criteria from the Oxford-Centre for Evidence Based Medicine.^13^ Primary outcomes assessed were the sensitivity and specificity of patients with ATFL injuries diagnosed via MRI compared to gold standard arthroscopic findings. Outcomes were compared between MRI in identifying ATFL instability with two evaulation strategies: discontinuity of the ligament alone versus discontinuity coupled with thickening and increased signal.

### Quality Appraisal and Risk of Bias

All included studies were assessed for risk of bias and study quality using the Methodological Index for Nonrandomized studies (MINORS) criteria.^14^ The MINORS criteria includes a 12-item checklist with each item receiving a score of either 0 (not reported), 1 (inadequately reported), or 2 (adequately reported). Noncomparative and comparative studies have a maximum score of 16 and 24 points, respectively. Table 1 summarizes the risk of bias.

**Table 1:** MINORS Criteria for Included Studies

MINORS Criteria	An et al. 2021^5^	Basha et al. 2021^16^	Brown et al. 2004^17^	Cardone et al. 1993^18^	Chan et al. 2013^19^	Farooki et al. 1998^20^	Haller et al. 2006^21^	Kim et al. 2015^9^	Kreitner et al. 1999^22^	Lee et al. 2012^23^	Morvan et al. 2018^24^	Verhaven et al. 1991^25^	Xu et al. 2021^3^	Yan et al. 2021^2^
A clearly stated aim	2	2	2	2	2	2	2	2	2	2	2	2	2	2
Inclusion of consecutive patients	2	2	2	2	2	2	2	2	2	2	2	2	2	2
Prospective collection of data	1	1	0	0	0	0	0	0	0	0	0	0	0	0
Endpoints appropriate to the aim of the study	2	2	2	2	2	2	2	2	2	2	2	2	2	2
Unbiased assessment of study endpoint	0	0	0	0	1	0	1	0	1	1	1	0	1	1
Follow-up period appropriate to the aim of the study	1	2	1	1	2	1	0	2	1	1	2	1	2	2
Loss to follow-up less than 5%	2	2	2	1	2	2	2	2	2	2	2	2	2	2
Prospective calculation of the study size	1	2	1	2	1	2	2	2	2	2	2	2	2	2
Total	11	13	10	10	12	11	11	10	10	12	13	11	13	13

2 = adequately reported; 1 = inadequately reported; 0 = not reported.

### Outcomes Analyzed and Statistics

All statistical analyses were performed using Review Manager (RevMan) (Macintosh; Version 5.3). The Nordic Cochrane Centre, The Cochrane Collaboration, 2014 and SAS version 9.4 (SAS Institute, Inc., Cary, NC). To compare diagnostic accuracy between discontinuity of the ligament alone versus discontinuity coupled with thickening and increased signal intensity, forest plots and summary receiver operating characteristic (sROC) curves were plotted by assessment strategy. A hierarchical sROC model was run using the metadas macro in SAS. Summary points including sensitivity, specificity, positive and negative likelihood ratios, and diagnostic odds ratios (OR) were summarized overall and by MRI assessment strategy with 95% confidence intervals (CIs). A *P*-value < .05 was considered to be statistically significant.

## Results

### Literature Selection

**Fig 1:**
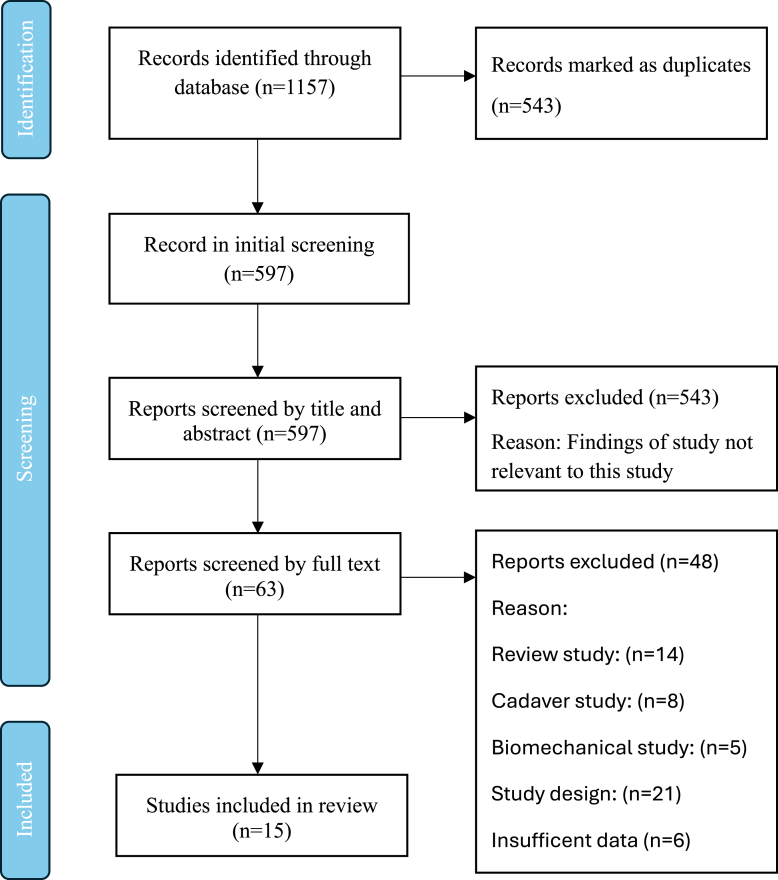
Overview of PRISMA Search. (PRISMA, Preferred Reporting Items for Systematic Review.)

The search yielded a total of 1,157 total citations, which were uploaded into Covidence for review.^15^ Covidence automatically removed 543 duplicate citations, leaving 597 unique citations for screening, and 9 studies were included.^2^^,^^3^^,^^5^^,^^9^^,^^16^, ^17^, ^18^, ^19^, ^20^, ^21^, ^22^, ^23^, ^24^, ^25^, ^26^ A visual representation of this process is illustrated in Figure 1.

A summary of 9 studies meeting inclusion and exclusion criteria for the systematic review are provided in Table 2. There were 15 studies that met our inclusion criteria but 6 studies had insufficient statistics to be included (i.e., no sensitivity and specificity values provided). Thus, data from 9 studies were ultimately included in the analyses (Fig 1).

**Table 2:** Patient Demographics and Study Characteristics

Study	Design	No. Patients	No. Ankles, Male/ No. Ankles, Female	Age, yr, mean ± SD (range)	How Long From Injury Was Imaging Taken, d, mean ± SD (range)	Level of Evidence
Basha et al. 2021^16^	Prospective	62	47/15	36.9 ± 12.1 (17-52)		II
Farooki et al. 1998^20^	Retrospective	12	7/5	38		III
Haller et al. 2006^21^	Prospective	51	31/20	36 (16-73)	5-10	II
Kim et al. 2015^9^	Retrospective	79	44/35	34.6 (21-67)	19.4 ± 9.8 (5-45)	III
Lee et al. 2012^23^	Retrospective	34	22/12	29 (13-53)	14 (1-84)	III
Morvan et al. 2018^24^	Retrospective	22	15/7	30.3 ± 9.5 (15-53)	83 ± 45.7 (23-159)	II
Verhaven et al. 1991^25^	Prospective	18	NR	21	Within 6 h	II
Xu et al. 2021^3^	Prospective	45	27/22	32.1 (18-58)		II
Yan et al. 2021^2^	Retrospective	158	94/64	33.0 (13-76)		III

NR, not reported; SD, standard deviation.

### Study Characteristics and Patient Demographics

This systematic review included a total of 9 studies featuring 481 patients with injuries to the ATFL. The MINORS criteria were used to evaluate the methodologic quality of the included studies. The average score for all of the studies was 12.4, signifying moderate-to-high quality of rigor.

Of 481 patients, there were 287 male and 180 female participants. The patients’ ages ranged from 9 to 79 years old, with an average of 32.7 years. There was an average follow-up time of 24.3 days from injury to imaging. Follow-up times varied greatly between studies. The longest recorded interval was more than 83 days, whereas the shortest was within 6 hours of injury (Table 2).

### Imaging and Diagnostic Accuracy

**Fig 2:**
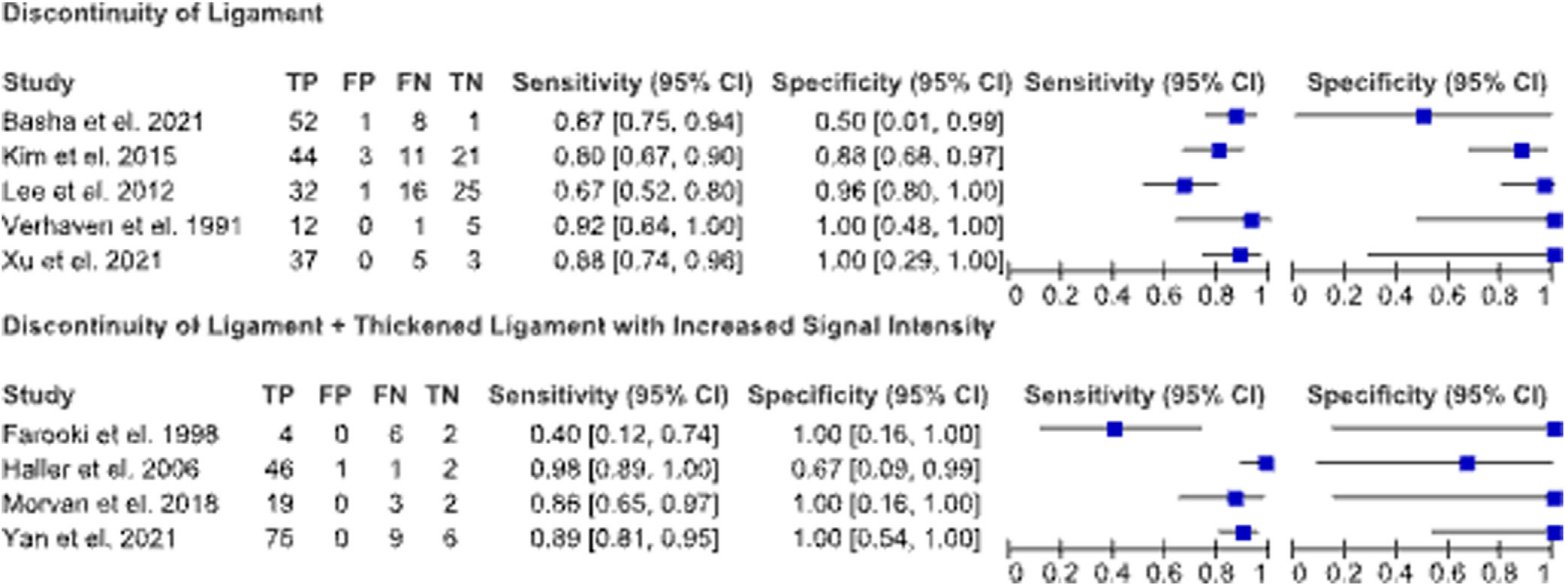
Sensitivity and specificity plots of MRI diagnosis. (CI, confidence interval; FN, false negative; FP, false positive; TN, true negative; TP, true positive.)

Table 3 summarizes the diagnostic properties of the studies included in this review. Table 4 summarizes the individual diagnostic accuracy for each individual study. The sensitivity of MRI for diagnosing ATFL injuries were relatively high across all studies compared to arthroscopic findings (Fig 2). Although specificity was also high, there were wide confidence intervals around the specificity values due to the small number of patients with no instability included in each of the studies. There was no evidence of a difference in sensitivity nor specificity between the two distinct MRI assessments of ligament discontinuity alone and coupled with ligament thickening with increased signal intensity (relative sensitivity, 1.03; 95% CI, 0.84-1.26; *P* = .79 and relative specificity, 1.08; 95% CI, 0.79-1.47; *P* = .62). The relative diagnostic odds ratio was high in both assessment types and not significantly different between groups (relative diagnostic odds ratio, 2.35; 95% CI, 0.17-31.9; *P* = .52).

**Table 3:** Summary of Model Results∗

	Discontinuity of Ligament	Discontinuity of Ligament + Thickened Ligament With Increased Signal Intensity	Comparison (Discontinuity of Ligament/ Discontinuity of Ligament + Thickened Ligament With Increased Signal Intensity)	*P* Value for Comparison
Sensitivity	84.7% (70.5-92.8%)	87.1% (67.3-95.7%)	1.03 (0.84-1.26)	.79
Specificity	84.6% (57.4-95.7%)	91.4% (45.7-99.3%)	1.08 (0.79-1.47)	.62
Positive likelihood ratio	5.5 (1.8-16.7)	10.1 (1.0-97.7)	−	−
Negative likelihood ratio	0.18 (0.10-0.33)	0.28 (0.01-11.23)	−	−
Diagnostic odds ratio	30.5 (10.4-89.8)	71.7 (6.4-808.5)	2.35 (0.17-31.9)	.52

∗ Data presented as mean (range).

**Table 4:** Diagnostic Accuracy for ATFL Injuries by Study

Study	Sensitivity (95% Cl)	Specificity (95% Cl)	Accuracy	Positive Predict Value (95% Cl)	Negative Predict Value (95% Cl)
Basha et al. 2021^16^	86.7%	50%	85.5%	98.1%	11.1%
Farooki et al. 1998^20^	42%	85%	69%		
Haller et al. 2006^21^	97%	53%			
Kim et al. 2015^9^	79.85%	87.5%	82.3%	65.8%	93.6%
Lee et al. 2012^23^	66.7%	97%	67.6%		92.3%
Morvan et al. 2018^24^	86.6%	89.8%		81.3%	92.9%
Verhaven et al. 1991^25^	100%	50%	94.40%		
Xu et al. 2021^3^	89%	100%	93%	100%	97%
Yan et al. 2021^2^	88.90%	100%	93.80%		

ATFL, anterior talofibular ligament; CI, confidence interval.

## Discussion

MRI proves high sensitivity and specificity in diagnosing ATFL injury. Using imaging characteristics such as ligament thickening and heightened signal intensity in conjunction with ligament discontinuity does not significantly increased diagnostic accuracy over ligament discontinuity alone.

Although many imaging modalities, including ultrasound, radiographs, and computed tomography, are used to assess ankle injuries, MRI is the preferred diagnostic imaging modality for ATFL injuries because of its superior soft-tissue resolution and multiplanar imaging capabilities.^27^, ^28^, ^29^ MRI not only provides detailed visualization of ligament integrity but also provides valuable information about associated conditions, such as bone marrow edema or concurrent ligament injuries, which may influence management strategies.^30^^,^^31^ Consequently, many physicians recommend obtaining an MRI before surgical interventions for CAI, because it offers high diagnostic accuracy and a non-invasive means of confirming suspected ligament disruptions.^8^^,^^32^

Our study’s high sensitivity and specificity values compared with arthroscopic findings align closely with other reported literature. Barini et al.^33^ demonstrated that MRI shows a pooled sensitivity of 100% and specificity of 90% for diagnosing acute ATFL injuries, emphasizing its utility in early-stage evaluation. This aligns with findings from Cao et al.,^8^ which reported MRI sensitivity and specificity compared with arthroscopic findings for chronic ATFL injuries at 83% and 79%, respectively. The reduced diagnostic accuracy in chronic cases may be attributed to morphologic changes such as scarring, thickening, or thinning that complicate MRI interpretation. These results emphasize the reliability of MRI for acute injuries. Chronically, the variability in ligament pathology hinders diagnostic clarity.^34^

Compared with arthroscopic findings, variations in the sensitivity and specificity across studies are likely the result of differences in imaging protocols, patient populations, and the timing of MRI relative to injury. All of the studies used a 1.5-Tesla MRI system, except Lee et al.^23^ and Xu et al.^3^ that used a 3.0-Tesla MRI system.^3^^,^^23^ Standardizing MRI protocols could help to achieve more reproducible and accurate results.^35^ Additionally, all the studies referenced arthroscopic or surgical findings as the gold standard of diagnosing ATFL injuries.^36^^,^^37^

Previous studies, such as those by Kim et al.^9^ and Lee et al.,^23^ have demonstrated the effectiveness of assessing ligament discontinuity alone for identifying instability, although variability in reported diagnostic accuracy has persisted. Our research incorporates an additional diagnostic marker in ligament thickening that may better capture the spectrum of injury pathology. This is advancement of previous research that didn’t include ligament thickening. Therefore, the potential role of ligament thickening as a MRI-based diagnostic parameter for ATFL injuries stimulates an important discussion for clinical decision-making. If ligament thickening were officially recognized as a key MRI marker for ATFL injury, treatment algorithms may be shifted toward earlier surgical intervention in order to decrease the risk of CAI and post-traumatic osteoarthritis (PTOA). According to recent studies, up to 78% of people with CAI develop PTOA when left untreated, highlighting CAI as a significant risk factor.^38^^,^^39^ The altered joint biomechanics and excessive joint loading is theorized to cause cartilage degeneration and narrowing of the joint space.^40^^,^^41^ MRI findings of ligament thickening, which were once thought to be a harmless adaptation, may signify degenerative changes, chronic stress-related hypertrophy, or failed healing, indicating a structurally compromised ligament that is unable to sustain joint stability.^10^^,^^42^ This supports early, aggressive surgical intervention to stabilize the joint and prevent irreversible long-term damage instead of conservative care.^39^ It has been suggested that nonsurgical therapy frequently fails to restore complete ligament integrity, causing laxity and dysfunctional biomechanics that feed the cycle of instability and joint deterioration,^41^ whereas early ligament repair has been shown to slow or stop the development of PTOA.^39^

Contrarily, some studies argue the sole importance of ligament discontinuity as the MRI measure for diagnosing ATFL injuries.^9^^,^^23^ This suggests that ligament thickening alone may not require surgical intervention. Furthermore, nonsurgical management can still be effective, as Altomare et al. identified that conservative management was noninferior to surgery when comparing various economic factors and rehabilitation outcomes.^43^^,^^44^ In addition, it is suggested that patients treated nonoperatively for ATFL injuries have higher satisfaction ratings and a shorter time to return to their work and leisure activities.^43^ For athletes, specifically, the incidence of re-injury, CAI, PTOA increases^45^^,^^46^; however, after surgery, some do not recover to preinjury levels.^45^

To balance these viewpoints while avoiding unnecessary surgery and optimizing ankle sprain treatment plans to reduce the long-term incidence of PTOA, an approach that includes the MRI criteria of ligament thickening and discontinuity appears favorable and could potentially enhance patient outcomes. Determining the need for surgical intervention should incorporate both ligament thickening and ligament discontinuity, as depicted by high signal intensity on MRI. Although our research revealed no statistically significant difference in diagnostic accuracy between these two methods, the coexistence of both findings can be suggestive of a structurally-compromised ligament and therefore pose a risk for CAI and PTOA. We also recommend surgical intervention if ligament thickening is seen on imaging because CAI is an established risk factor for PTOA,^45^^,^^46^ MRI evidence of progressive structural deterioration, such as complete ligament discontinuity, significant thickening with changed signal intensity, and joint instability, can be an indication for early surgical intervention. Although our review did not demonstrate statistically significant difference in diagnostic accuracy between the 2 methods, we do recommend surgical intervention if ligament thickening is present as this is associated with CAI, and ultimately PTOA. Further longitudinal research is needed to better understand and evaluate the independence and interplay of ligament thickening and discontinuity prognostic values to refine ATFL injury algorithms for operative versus nonoperative management.

### Limitations

As a systematic review, this study has several limitations and potential bias sources. Many of the included studies have their own set of limitations. Some studies failed to report critical data, which could impact the accuracy and completeness of the analysis. In addition, all the studies were cohort studies that lacked randomization, introducing potential biases that could affect the validity of their results. The lack of randomization in these studies limits the ability to establish causal relationships by raising questions about confounding variables that could affect the observed outcomes. There were differences in the MRI protocols used, which could have led to a discrepancy in the values. Another limitation is having a limited number of studies included. There were 15 studies that met the inclusion criteria but only 9 studies were included. Out of the nine there were only 2 studies that represented CAI. There were also only 4 studies that included ligament thickening. These results may not represent a large population due to the small sample size. None of the included studies disaggregate data by sex to determine its impact. Several studies reported percentage of male and female participants; none analyzed diagnostic performance. Future studies should consider sex in analyses in accordance with SAGER (Sex and Gender Equity in Research) guidelines.

## Conclusions

This study found that incorporating ligament thickening and increased signal intensity alongside ligament discontinuity on MRI does not significantly improve diagnostic accuracy.

## Disclosures

The authors declare the following financial interests/personal relationships which may be considered as potential competing interests: D.B. reports support was provided by Duke University School of Medicine. C.G. reports support was provided by Duke University School of Medicine S.H. reports support was provided by Duke University School of Medicine. B.L. reports support was provided by Duke University School of Medicine. E.P. support was provided by Duke University School of Medicine. J.R. reports support was provided by Duke University School of Medicine. J.T. reports support was provided by The University of North Carolina at Chapel Hill School of Medicine. A.T.A reports support was provided by Duke University Department of Orthopaedic Surgery.
